# Long-term follow-up after introduction of a systematic sexually transmitted infection screening program for men having sex with men living with HIV in a primary care setting: uptake, STI incidence, and risk factors for infection and reinfection

**DOI:** 10.1007/s15010-022-01946-0

**Published:** 2022-11-09

**Authors:** Philipp J. G. Mathé, Susanne Usadel, Siegbert Rieg, Winfried V. Kern, Matthias C. Müller

**Affiliations:** 1 Division of Infectious Diseases, Department of Medicine II, Medical Center, Faculty of Medicine, University of Freiburg, Hugstetter Straße 55, 79106 Freiburg, Germany; 2 Department of Infection Medicine, Medical Service Centre Clotten, Freiburg, Germany

**Keywords:** STI incidence, STI screening, HIV, MSM, Primary care

## Abstract

**Purpose:**

Annual screening for asymptomatic infections with *Chlamydia trachomatis* (CT) and *Neisseria gonorrhoeae* (NG) is recommended by international guidelines in people living with HIV but uptake in routine care remains poor. This study analyzed the effects of the implementation of a CT/NG screening program in a primary HIV treatment center.

**Methods:**

In this single-center cohort study, we included men having sex with men (MSM) living with HIV during the study period from January 2016 to December 2019. From January 2018 on, annual sexual health counseling including CT/NG screening was proactively offered to all MSM presenting at the center. CT/NG screening rates, test positivity rates and case detection rates in the years 2018 and 2019 were compared to those in the years 2016 and 2017.

**Results:**

A total of 234 patients were enrolled in the study contributing to 798.7 patient years (py) during the four-year study period. Screening rates increased from 3.1% and 3.9% in 2016 and 2017 to 51.1% in 2018 and decrease to 35.4% in 2019. Over the study period, 19.7% (46/234) had at least one positive CT/NG result. After the intervention, case detection per 100 py increased for CT (2016: 2.6, 2017: 3.7, 2018: 7.7, 2019: 7.1) and NG (2016: 3.2, 2017: 3.1, 2018: 5.3, 2019: 7.6). The number needed to test was 8.9 for CT and 10.4 for NG.

**Conclusion:**

Regular CT/NG screening is feasible in a primary care setting, leads to an increase in case detection and may contribute to decrease transmission and complications of CT/NG.

**Trial registration:**

The trial is registered in ClinicalTrials.gov (NCT02149004).

**Supplementary Information:**

The online version contains supplementary material available at 10.1007/s15010-022-01946-0.

## Introduction

The increasing incidence of sexually transmitted infections (STIs) is a growing problem in men having sex with men (MSM), especially in those being infected with HIV, [1–6]. Epidemiological data from 2018 and 2020 of several large cities in Germany showed high prevalence for infection with *Chlamydia trachomatis* (8.8–9.9%), *Neisseria gonorrhoeae* (6.8–8.9%) and for Syphilis (1.4%) in MSM with HIV-infection, most of them reporting no symptoms (68–91%) [3, 7, 8]. The often asymptomatic course is a hallmark of many STIs; hence, a significant proportion of STIs remain undetected and serve as a reservoir for further transmission. Moreover, untreated STIs may eventually result in serious long-term sequelae. Thus, implementation of annual STI screening tests for asymptomatic STIs in MSM with HIV-infection is recommended by European guidelines as they may be beneficial for patients’ health, block transmission chains, and reduce STI prevalence [9–14].

Whereas data are lacking in Germany, studies from UK, USA, and Canada suggest that STI testing uptake in HIV cohorts remains suboptimal, especially in MSM [15–18]. In a multicenter study in several HIV cohorts in USA, annual testing rate for NG/CT was only 39% of patients engaged in care compared to 77% testing rate for syphilis, although most patients reported sexual behavior with a higher possibility for STI transmission [17].

Increasing rates of CT/NG testing in HIV clinics may be achieved by reduction of barriers at the clinician, patient, and system level [19]. Much of the above-mentioned contrast between syphilis and CT/NG testing coverage may stem from physician’s lack of time and discomfort with sexual history taking and collection of STI swabs [20, 21]. A survey analyzing the patients’ satisfaction with sexual and reproductive health (SRH) services in HIV primary care identified shortage of time, a lack of service providers’ initiative, and patients’ difficulty to address SRH topics as most relevant hindering factors for satisfying SRH services [22]. Structural or systems level interventions appear to be more effective and sustainable to improve STI screening rates than interventions on patients or clinicians level [23]. Strategic placement of specimen collection materials or automatic collection of STI specimens as part of a routine visit, the use of electronic health reminders for providers and patient reminders for screening or rescreening were among the most effective tools to enhance rates of STI testing [23]. However, effect of interventions may differ depending on the targeted population and the setting. In the setting of primary HIV care, patients are generally closely followed up over years with several annual routine appointments allowing high quality patient-provider relationships. To our knowledge, only two studies evaluated the effect of a system level intervention to increase CT/NG in the setting of HIV care [24, 25]. Introducing a combined annual anal cytology and CT/NG screening program was associated with an increased CT/NG testing rate during the short-term follow-up of 3 months.

In this study, we report the long-term effects of a system level intervention to increase CT/NG screening in a primary HIV care clinic in a mid-size city in Germany. Up from January 2018, patients were offered annual physician initiated sexual health counseling including syphilis and CT/NG screening and instructions for future low-threshold, self-collected CT/NG testing at patient’s discretion. Rates of CT/NG screening, CT/NG test positivity, and CT/NG diagnosis in a period of 2 years post implementation were compared to a 2-year pre-implementation period.

## Methods

### Ethics statement

The study received ethics approval from the institutional review board of the University Medical Center of the University of Freiburg (No.: 469/17/Date: 10. Oct. 2017). All PLWH were recruited in an outpatient care setting in Freiburg and provided written informed consent.

### Study site/setting

This is a single-center, retrospective, longitudinal cohort study comparing a pre-intervention (01/2016–12/2017) and intervention period (01/2018–12/2019). The study took place at a specialized HIV primary care center caring for about 900 adult patients living with HIV situated in Freiburg, a regional metropolis in the south west of Germany (population of the region 2016: 2,239,734 [26]). Clinically stable patients generally present once per quarter for routine follow-up including medical history, directed physical exam, laboratory testing, and drug supply.

All HIV seropositive MSM > 18 years presenting between January 2018 and December 2019 having provided informed consent were enrolled in the study, and data were subsequently extracted from their electronic medical records. For the calculation of the months with viral load below the level of detection (VLbLOD), a blip of viremia was allowed with once detectable HIV-RNA up to 200 copies/ml [27]. Relationship status was determined by the number of sexual partners and the presence of one stable partner (changing partner = multiple sexual partners, no stable one; open relationship = multiple sexual partners, but one predominantly partner; monogamous = one stable sexual partner; group sex = frequent multiple sexual partners at the same time).

Patients were grouped in two groups according if they had a positive tests for CT or NG over the whole study period. The rate of positive tests was calculated per one hundred patient years summarizing the results of all patients counted for every year of inclusion. To assess the effort for receiving positive test results, we calculated a number needed to test according to the following formula:

$${\text{Number}}\,{\text{ needed }}\,{\text{to}}\,{\text{ test}}\, = \,\frac{1}{{{\text{estimated}}\,{\text{ risk}}\,{\text{ of}}\,{\text{ a }}\,{\text{positive}}\,{\text{ result}}}} = \frac{1}{{\left( {{\text{proportion}}\,{\text{ of}}\,{\text{ positive}}\,{\text{ results}}\,{\text{ in}}\,{\text{ the}}\, \left( {\text{sub - }} \right)\,{\text{group}}\,{\text{ of}}\,{\text{ interest}}} \right)}}$$

### Intervention

From January 2018 on, all patients were assigned to an intervention consisting in a prolonged consultation of 30 min including among others screening for risk of renal, bone, cardiovascular disease [28]. Additionally, all MSM were offered a semi-structured interview assessing relationship status (monogamous vs. changing partner vs. group sex vs. open relationship), sexual practices (active vs. not active anal sex), and condom use (always vs. not always vs. no) as well as STI screening consisting of syphilis serology (TPHA, confirmed by immunoblot; VDRL as activity parameter) and pooled nucleic acid amplification tests (NAAT) for CT and NG from oropharyngeal, urethral, and anorectal swabs. MSM were introduced to self-collection of samples and encouraged to ask for STI screening at their discretion. Clinician initiated sexual health counseling and STI testing was offered on an annual basis. Before 2018 STI counseling and testing was offered at clinician discretion depending on the sexual history or patient’s initiative and not included in a structural sexual health assessment. The included cases in the pre-screening era were asymptomatic. In case of a positive test result, the corresponding patient was informed and treated according to the respective guideline.

### Statistical analysis

For assessment of differences between sociodemographic, behavioral, and HIV-specific factors, Fisher’s exact test or Chi-square test for categorical variables and Mann–Whitney *U* test for continuous variables were used to detect differences between groups. To further investigate the association between different factors and the risk of a positive STI test, a multivariable binominal logistic regression model was calculated. The outcome variable was defined as “Having at least one positive test for CT or NG” vs. “Having no positive test for CT or NG.” Variables were included showing a significant difference in a univariate binominal logistic regression. More than 10 observations per independent variable could be included and collinearity was checked via Variance Inflation Factor. To control for differences in the frequency of testing between different patients, the number of tests was included as a covariate term.

Differences between the pre-intervention time period (2016 and 2017) and the intervention time period (2018 and 2019) were assessed summarizing these periods for the average positive test count per quartal and analyzed by univariate linear regression. This was done to identify trends in the rate of positive tests which may be explained by underlying changes in STI incidence.

*p* values < 0.05 were considered to show statistical significance. All data analyses were done with pseudonymized data using R and R Studio (version 4.0.2) [29].

## Results

### Cohort characteristics

Our recruited cohort was comprised of 235 MSM living with HIV, whereat one patient was excluded because of missing data. In total of 234 MSM living with HIV contributed to 798.7 patient years (py) during the 4-year study period. Patients were followed up for a median of 4 years (Min–Max: 0.1–4.0 years). The median age at enrollment was 47 years (1.Q.–3.Q: 38–53 years), median time since HIV diagnosis was 6 years (1.Q.–3.Q: 3–11 years), and all patients but one were on ART since a median of 3 years (1.Q.–3.Q: 1–5 years, Table 1).

**Table 1:** Characteristics of participants of the study evaluating introduction of structured STI screenings in men having sex with men in a German HIV cohort between 01/2016 and 12/2020

	Total	Patient with no CT/NG infection	Patients with one pos. CT/NG testing visit			Patients with recurrent pos. CT/NG testing visits		
				OR [95%-CI]	*p* value		OR [95%-CI]	*p* value
*n*	234	186	30			18		
Age (y), Median (1.Q.-3.Q.)	47 (38.0–53.0)	47 (39.0–54.0)	43 (33.5–48.8)		0.558^a^	42 (35.3–44.8)		**0.003**^a^
Included patient years	798.7	627.5	101.8			65.4		
Time since HIV diagnosis (y), Median (1.Q.–3.Q.)	6 (3–11)	6 (4–13)	4 (2–7.3)		0.114^a^	3 (1.3–5)		**0.009**^a^
ART use (%)	233 (99.6%)	185 (99.5%)	30 (100%)			16 (100%)		
Time since ART start (y), Median (1.Q.–3.Q.)	3 (1–5)	3 (2–5)	3 (1.3–3.8)		0.087^a^	1 (0.5–2.5)		**0.007**^a^
CD4 nadir (cells/µl), Median (1.Q.–3.Q.)	303 (172.5–452.0)	290 (164.5–438.2)	333 (162.5–491.0)		0.472^a^	413.5 (367.2–530)		**0.002**^a^
CD4, (cells/µl), Median (1.Q.–3.Q.)	691.5 (505.2–917.2)	656 (489.0- 890.0)	770.5 (568–911.8)		0.679^a^	817.5 (757.2–1154.2)		**0.004**^a^
HIV-RNA load above LOD, *n* (%)	3 (1.3%)	3 (1.6%)	0 (0%)	NA	1^b^	0 (0%)	NA	1^b^
VLbLoD (months), Median (1.Q.–3.Q.)	48.0 (26.0–84.0)	52.0 (26.0–86.0)	42.5 (24.3–76.5)		0.088^a^	47 (32–49)		0.235^a^
Condom use, *n* (%)								
*Always*	45 (19.2%)	32 (19.9%)	8 (26.7%)	1.4 [0.4–4.2]	0.604^b^	5 (27.8%)	1.3 [0.3–5.3]	0.751^b^
*Sometimes*	25 (10.7%)	18 (8.8%)	3 (10.0%)	0.8 [0.1–3.1]	1^b^	4 (22.2%)	2.0 [0.4–8.7]	0.275^b^
*No*	53 (22.6%)	42 (23.5%)	8 (26.7%)	0.9 [0.3–2.6]	0.806^b^	3 (16.7%)	0.4 [0.1–1.7]	0.224^b^
*Unknown*	111 (47.4%)	93 (50.3%)	11 (36.7%)			6 (33.3%)		
Sexual behavior, *n* (%)								
* Monogamous*	81 (34.6%)	75 (32.1%)	4 (13.3%)	0.2 [0.1–0.7]	**0.005**^b^	2 (11.1%)	0.2 [0–0.8]	**0.010**^b^
* Open relationship*	49 (20.9%)	35 (15.0%)	9 (30.0%)	1.8 [0.7–4.7]	0.219^b^	5 (27.8%)	1.6 [0.4–5.1]	0.375^b^
* Changing partners*	50 (21.4%)	29 (12.4%)	13 (43.3%)	4.1 [1.6–10.3]	**0.002**^b^	8 (44.4%)	4.1 [1.3–12.6]	**0.008**
* Group sex*	1 (0.4%)	1 (0.4%)	0 (0%)	NA	1^b^	0 (0%)	NA	1^b^
* No relationships*	36 (15.4%)	29 (12.4%)	3 (10.0%)	0.6 [0.1–2.1]	0.584^b^	3 (16.7%)	1.0 [0.2–3.8]	1^b^
* Unknown*	8 (3.4%)	8 (3.4%)	0 (0%)	NA	0.604^b^	0 (0%)	NA	1^b^
Active hepatitis C, *n* (%)	2 (0.9%)	2 (0.9%)	0 (0%)	NA	1^b^	0 (0%)	NA	1^b^
Number of STIs per 100py	16.5	4.8	43.2			88.6		
Number of CT or NG tests per 100py	42.9	28.1	72.7			142.1		
Number of CT per 100py	5.3	0	20.6			33.6		
Number of NG per 100py	4.9	0	11.8			41.3		
Number of syphilis tests per 100py	253.6	258.0	300.6			293.4		
Number of aTP per 100py	5.9	4.8	10.8			9.2		

Odds ratios were calculated in comparison of the group to patients with no positive testing visit for CT or NG. Bold figures mark significant *p* values in the performed group comparison

*ART* anti-retroviral therapy, *aTP *active *Treponema pallidum* infection, *CT* *Chlamydia trachomatis* infection, *IQR* interquartile range, *NG* *Neisseria gonorrhea* infection, *Pos* positive, *py* patient years, *STI* sexually transmitted infection, *VLbLoD *viral load below limit of detection, *y* year

^a^Group differences tested by Mann–Whitney *U* test

^b^Group differences tested by Fisher’s exact test

### Detection of *C. trachomatis*, *N. gonorrhoeae*, and active syphilis

Positive results for CT were found in 12.8% (43/335) of all CT screening tests and 13.2% (31/234) of all patients during the study period between January 2016 and December 2019 (Tables 1 and 2). The rate of positive results for NG was 11.4% (39/341) of all NG screening tests and 10.7% (25/234) of all patients during the whole 4 years. Therefore, regarding CT 5.4 positive tests per one hundred patient years were found, quite similar to the rate of NG with 4.9 positive tests per one hundred patient years. Active *Treponema pallidum* infection (aTP) was diagnosed in 14.1% (33/234) of patients during the 4 years resulting in 5.9 aTP cases per one hundred patient years. Of all included patients, 23.1% (54/234) had at least once a positive test result for CT, NG, or aTP infection unnoted of the testing indication.

**Table 2:** Introducing low-threshold screening for *Chlamydia trachomatis* and *Neisseria gonorrhoeae* in men having sex with men in a German HIV cohort

	2016	2017		2018	2019	2016–2019	*p* value^a^
N	193	205	Start Intervention January 2018	221	223	234	
Patient years	189.58	190.57		206.67	211.91	798.6 (792.3)	
CT/NG screenings (*n*)	7	8		127	89	231	
CT/NG screenings/100 patient years (*n*)	3.7	4.2		61.5	42.0	28.9	
CT/NG screening at least once (%)	3.1 (6/193)	3.9 (8/205)		51.1 (113/221)	35.4 (79/223)	62.0 (145/234)	** < 0.001**
Screening for syphilis at least once (%)	99.0 (191/193)	99.5 (204/205)		99.1 (219/221)	100 (223/223)	97.9 (229/234)	1
CT cases	5	7		16	15	43	
CT test positivity rate (%)	20.0 (5/25)	21.9 (7/32)		10.5 (16/152)	11.8 (15/127)	12.8 (43/337)	0.069
Proportion diagnosed with CT (%)	2.1 (4/193)	3.6 (7/197)		6.8 (15/221)	5.4 (12/223)	13.2 (31/234)	**0.036**
CT cases per 100 py	2.6	3.7		7.7	7.1	5.4	
NG cases	6	6		11	16	39	
NG test positivity rate (%)	23.1 (6/26)	18.2 (6/33)		7.1 (11/154)	12.6 (16/127)	11.4 (39/341)	**0.034**
Proportion diagnosed with NG (%)	2.6 (5/193)	2.5 (5/197)		4.5 (10/221)	5.8 (13/223)	10.7 (25/234)	0.062
NG cases per 100 py	3.2	3.1		5.3	7.6	4.9	
Number of aTP screenings/ 100py	225.2	249.8		243.4	351.6	269.3	
aTP cases	14	12		8	12	47	
aTP test positivity rate (%)	3.2 (14/437)	2.5 (12/489)		1.4 (8/552)	1.5 (12/778)	2.1 (47/2227)	0.055
Proportion diagnosed with aTP (%)	5.2 (10/193)	5.4 (11/205)		3.6 (8/221)	5.4 (12/223)	14.1 (33/234)	0.553
aTP cases per 100 py	7.4	6.3		3.9	5.7	5.9	

*aTP*  active *Treponema pallidum* infection, *CT* *Chlamydia trachomatis* infection, *NG* *Neisseria gonorrhea* infection, *Pos* positive, *py* patient years

^a^Differences between pre-screening (2016/2017) and screening period (2018/2019) tested by Chi-square test. Bold figures mark significant *p* values in the performed comparison

### Effect of intervention on frequency of *C. trachomatis* and *N. gonorrhoeae* screening

In all, 62.0% of patients underwent screening for CT and NG at least once between 2016 and 2019 (Table 2, Supplement Fig. 1). After implementation of the intervention in January 2018, the proportion of yearly tested patients sharply increased from 3.1% in 2016 and 3.9% in 2017 to 51.1% in 2018 (*p < *0.001) and decrease to 35.4% in 2019. There was a continuously high proportion of patients screened for syphilis throughout the whole study period.

### Effect of the intervention on detection of *C. trachomatis*, *N. gonorrhoeae*, and active syphilis

After the implementation of the intervention, the positivity rates for CT and NG were approximately bisected, whereas the proportion of positive tested patients roughly doubled (Table 2). The rate of positive tests for aTP and the proportion of positive tested patients dropped after the intervention).

**Fig. 1:**
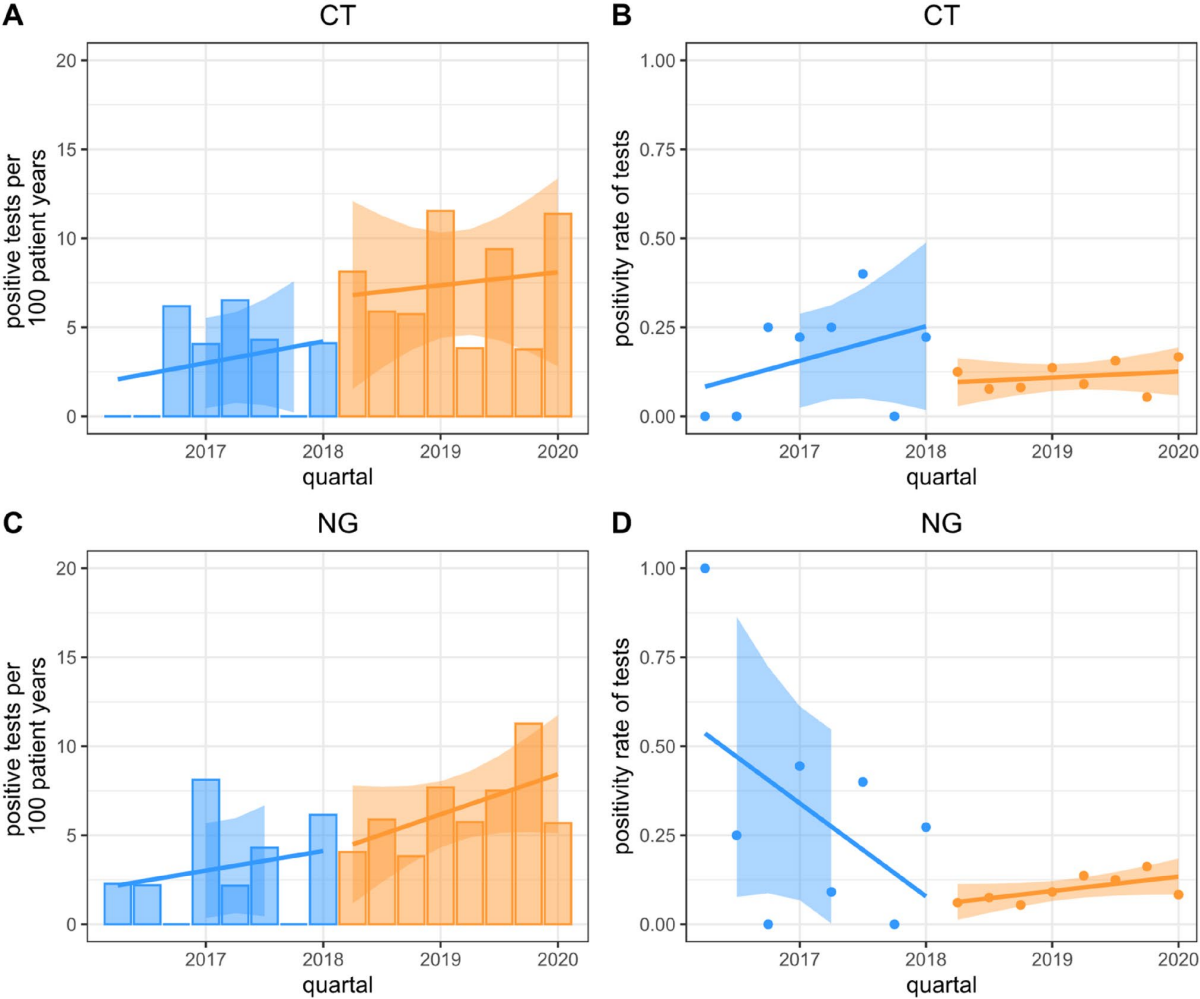
Number of positive tests and test positivity rate by quartal. The figure shows the proportion of positive tests [**A**: *Chlamydia trachomatis* (CT), **C**: *Neisseria gonorrhoeae* (NG)] and the total number of positive test per 100 person years [**B**: *Chlamydia trachomatis* (CT), **D**: *Neisseria gonorrhoeae* (NG)] by quartal before (blue) and after (orange) introducing of a low-threshold STI screening in men having sex with men in a German HIV cohort

We also checked for a background trend by univariate linear regression of increasing number of the different STIs by quartal, but no significant increment of positive tests was found for the period before the start of the screening and after. The rate of positive tests before and after the regular screening start is shown in Fig. 1.

We further calculated the number needed to test (NNT) to detect one case of an asymptomatic STI after the start of the screening. NNT was 8.9, 10.4, and 47.6 for one case of CT, NG, and aTP, respectively.

### Whom to screen?

In the following, we compare patients with at least one positive test result for an infection by CT or NG to those with no detected infection (Table 1). Here we found a significant higher proportion of patients having changing sexual partners (*p = *0.010) and a significant lower proportion having a monogamous relationship (*p = *0.015). While this was also the case for patient with recurrent infection (changing partners *p = *0.002, not monogamous *p = *0.001), for aTP infections no significant differences could be identified.

To identify risk factors for having at least one positive screening test for CT or NG out of a set of eligible variables, we performed a univariate logistic regression analysis and subsequently a multivariate logistic regression (Table 3). Significant univariate predictors for at least one positive test result included in the multivariate regression were time living with HIV (OR 1.08; *p = *0.007), having changing sexual partners (OR 4.00; *p < *0.001), being between 30 and 40 years old (OR 0.43; *p = *0.012) and living in a monogamous relationship (OR 0.19; *p < *0.001). Overall, the model (Nagelkerke *R*^2^ = 0.606) showed only a poor improvement over the baseline model with only the number of performed tests for CT or NG (Likelihood ratio test *p = *0.2815) and therefore no clear defined risk groups. ROC curve analysis for different cut-off points of numerical variables like age, CD4 count or time since HIV diagnosis also showed no favorable cut-off with a sensitivity of about 75% combined with a specificity of 50%.

**Table 3:** Logistic regression analysis of patients with at least one infection by CT/NG versus patients with no CT/NG infection after introduction of structured STI screenings in men having sex with men in a German HIV cohort between 01/2016 and 12/2020

Explanatory variable	Univariate regression		Multivariate regression				
	OR (95% CI)	*p* value	*β*	SE *β*	Wald’s *χ*^2^	*p* value	aOR (95% CI)
Intercept			**4.41**	**1.00**		** < 0.001**	
HIV characteristics							
Present CD4 count [cells/0.1 cm^3^]	0.91 (0.84–1.00)	0.061					
Years since diagnosis with HIV	**1.08 (1.03–1.14)**	**0.007**	0.02	0.04	0.17	0.682	1.02 (1.00–1.09)
Years since start of ART	1.11 (1.01–1.23)	0.088					
VLbLOD	1.00 (1.00–1.01)	0.599					
HIV viral load over LOD	NA	0.986					
Sexual behavior							
*Changing partner*	**4.00 (2.21–7.13)**	** < 0.001**	0.51	0.53	0.91	0.339	1.66 (0.69–4.00)
*Open relationship*	1.67 (0.90–3.04)	0.165					
* Monogamous*	**0.19 (0.08–0.40)**	** < 0.001**	-0.81	0.69	1.39	0.238	0.45 (0.13–1.32)
Use of condoms							
*Always*	1.35 (0.67–2.72)	0.475					
*Sometimes*	1.20 (0.51–2.69)	0.718					
* No*	0.65 (0.32–1.31)	0.324					
Age groups							
* 20–30*	2.17 (0.49–21.92)	0.470					
* 30–40*	**0.43 (0.24–0.75)**	**0.012**	− 0.64	0.52	1.53	0.216	0.45 (0.22–1.24)
* 40–50*	0.56 (0.33–0.96)	0.078					
Number of included years	0.90 (0.69–1.17)	0.547					
Number of CT/NG tests	**0.50 (0.42–0.58)**	** < 0.001**					**0.53 (0.44–0.62)**

AIC = 129.51, Cox and Snell *R*^2^ = 0.388, Nagelkerke *R*^2^ = 0.606. Overall model evaluation: Likelihood ratio test (*df* = 4, *χ*^*2*^ = 5.0572, *p < *0.2815), Wald test (*df* = 4, *χ*^*2*^ = 4.689, *p = *0.3207), Score test (*df* = 4, *χ*^*2*^ = 4.9408, *p < *0.2934). Outcome variable (Having at least one positive testing visit for *CT* or *NG* = 1/high risk group; Having no positive testing visit for *CT* or *NG* = 0/low risk group). Figures in bold indicate significant *p* values < 0.05 in the univariate logistic regression. Group sex was excluded because complete separation happened. Condom use was excluded because of many missing values to build the model on as many cases as possible. The multivariate model included all significant variables in the univariate regression. Adjusted OR were calculated including “Number of CT/NG/TP testing visits” in the logistic regression model

*aOR* adjusted odds ratio, *ART* anti-retroviral therapy, *aTP*  active *Treponema pallidum* infection, *CI* confidence interval, *CT* *Chlamydia trachomatis*, *df* degrees of freedom, *LOD* level of detection, *NG* *Neisseria gonorrhea*, *OR* odds ratio, *SE* standard error, *STI* sexually transmitted infection, *VLbLOD* Months viral load below level of detection

### Whom to re-screen repeatedly, and when?

With 7.7% (18/234) a significant proportion of the cohort was tested repeatedly positive by CT/NG screening during the study period. In total, 12 cases in 8 patients of repeated positive CT screening tests and 14 cases in 11 patients for NG were found, compared to 14 cases of aTP in 11 patients, despite more tests for aTP. Nevertheless, the larger proportion of positive patients were positive only once for any STI (53.0% (35/66)), but still a large proportion of positive tests were caused by patients with multiple positive screening tests (25.8% of all CT (8/31), 40.0% of all NG (10/25) and 33.3% of all aTP (11/33)) positive patients). The median time between two positive tests of all pathogens was about 279 days (1.Q–3.Q.: 120–457 days), higher for two positive tests for CT (median difference = 317 days), a bit lower for NG tests (median difference = 270.5 days) and much higher for aTP tests (median difference = 405 days). Differentiated for subgroups the median time between two positives tests for any STI time points varied widely for different sexual behavior (changing partners = 161 days [1.Q.–3.Q.: 77–384 days], open relationship = 309 days [1.Q.–3.Q.: 125–409.8 days] monogamous = 397 days [1.Q.–3.Q.: 167.5–886.8 days]) but were quite stable for age groups (30–40 years: 298 days [1.Q.–3.Q.: 91–397 days], 40–50 years: 281 days [1.Q.–3.Q.: 119–495.5 days], ≥ 50 years: 270.5 days [1.Q.–3.Q.: 223.5–395.5 days]).

To identify patient characteristics that could help to test patients with a high risk of having repeated STIs, we performed a second univariate logistic regression analysis with subsequent multivariate logistic regression (not shown in detail). Significant predictors for repeated positive testing visits in the multivariate logistic regression were only the time on ART (aOR = 0.47; *p = *0.0417) and the number of performed CT/NG tests (aOR 2.05; *p = *0.0003). Overall, the model showed a moderate fit (Nagelkerke *R*^2^ = 0.706, Likelihood ratio test *p = *0.0040).

## Discussion

Introducing periodic sexual health counseling and low-threshold, self-collected CT/NG screening in a primary HIV care setting showed sustained increasing CT/NG testing rates resulting in a sustained increase in case detection despite a drop in CT/NG test positivity. The proportion of patients with CT or NG were slightly higher compared to other cross-sectional studies, but comparable to one Irish study [3, 7, 8, 30]. To our best knowledge, this is currently the largest study evaluating the long-term effect of a systemic intervention to increase CT/NG screening rates in the setting of primary HIV care.

Since MSM with HIV are often exposed to discrimination and stigmatization and sexual health topics still represent a taboo issue, the use of STI related health care by MSM is still effectively influenced by shame and fear of homophobic reactions [31]. In our intervention, providers proactively addressed sexual health in a stigma-free approach creating a supportive environment, which may have encouraged many patients to openly discuss their issues and claim sexual health diagnostics. In addition, introducing and promoting of self-collection of swabs may have substantially contributed to the increase in CT/NG screening rates as it reduces workload for providers and enhances patient comfort and autonomy. Self-collected specimens show comparable sensitivity compared to provider-collected swabs [32, 33]. Our results show that this intervention on a system level yielded a substantial and persistent increase in CT/NG screening rates in the setting of primary HIV care. Interestingly, CT/NG screening rates dropped in the second year after the introduction of the intervention, while syphilis screening rate remained high. Reduced testing is probably explained by patient-perceived lower risk of infection in the follow-up testing. Indeed, especially in NG, case detection and proportion of positive tested patients increased in the second year. The long-term screening rate of 35.4% achieved in our study is in line with those published in other studies of 32–39% [15, 18, 34] and may cover most of the 42% of the population at risk of STI stating living in an open partnership or having changing partners.

Concerns exist that increased CT/NG testing rates may be countered by declines in NG/CT test positivity failing to increase overall CT/NG case detection [18, 34]. Our study showed a relevant decline in test positivity rates and increased case detection in the MSM cohort confirming results from Berry et al. showing increased case detection in the entire HIV cohort but most pronounced in the subgroup of MSM. The selection of asymptomatic patients tested in the pre-screening era was mainly driven by the clinicians discretion, which resulted in a higher test positivity rate but presumably in a less higher detection rate of CT/NG cases. At the same time, the screening program showed a quite high efficiency with low number of patients needed to screen to find one CT/NG case. Efficiency could further be increased focusing on MSM at elevated risk allowing reducing costs and workload. In line with previous studies, we identified a more recent diagnosis of HIV-infection, higher CD4 count, and changing sexual partners as predictors of asymptomatic STI [34–36].

Patients with CT or NG infection more often showed a higher CD4 count even with a lower time on ART, maybe reflecting a “healthier” state and therefore maybe a sexual more active part of our population. But previous studies also showed a higher rate of disclosure of HIV status to secondary partners if the CD4 count is known [37]. The association with positive tests could probably be explained by a higher level of “perceived security” by the patients if having higher cell counts and subsequently impaired protection measures, highlighting the importance of differentiated education about other sexual health risks.

Limiting CT/NG screening to MSM with arbitrarily set cutoffs with, e.g., CD4 > 350, age < 40 years, time since HIV diagnosis < 6 years would have missed a substantial part of positive cases. In addition, even patients denying sexual activities were at risk of both infection and reinfection, questioning the reliability of sexual history obtained in the clinic setting. Limiting screening to patients depending only on their sexual history could therefore be misguiding. Thus, considering the high CT/NG incidence and the increased case detection after introduction of the intervention, a broad approach of annual CT/NG screening as recommended in European guidelines seems to be reasonable at least in MSM [14].

### Limitations

No update on demographical data and information about sexual practice were available during the whole 4 years. Therefore, changes in the reported behavior or clinical information could have happened with an important impact on the risk for acquiring a STI, like breaking up monogamous relationship and now have changing partners or non-compliance to medication intake with a higher viral load and thereby higher immunosuppression. However, reports on sexual activity in Germany showed a quite stable sexual behavior over longer time periods [38]. Due to the retrospective study design including all MSM > 18 who presented from 01/2018 to 12/2019 at the center having provided broad informed consent for retrospective data analysis, selection bias appears unlikely, but cannot be fully excluded. Moreover, even if we have seen no clear background trend between the two study phases suggesting a significant change in STI incidence, a changing baseline incidence of STIs cannot be excluded. A decline in the number of STIs by identification and treatment could be counterbalanced by an overall increase in STI incidence and therefore a higher rate of reinfection [1].

Nevertheless, we are reporting real-world data with real-world challenges: this intervention took place in a busy primary HIV care service on a voluntary basis not restricted by study protocols. This resulted in a fluctuating adherence to the screening frequency as previously reported [39], but shows also the performance and possibilities for such screening programs in the real-world setting.

## Conclusion

In summary, we could show that CT/NG screening is feasible in a primary HIV care setting yielding a considerable high incidence of STIs with a low number needed to test in our population of MSM living with HIV. Routine CT/NG screening in vulnerable populations in primary HIV care might be an important contribution to tackle rising STI incidence in Germany and elsewhere. This screening model was only evaluated for the group of MSM living with HIV. Evaluation in and extension to other vulnerable groups, like commercial sex workers, seems warranted.

## Supplementary Information

Below is the link to the electronic supplementary material.

Supplementary file1 (DOCX 950 kb)

## Data Availability

Data are available upon request.

## Abbreviations

100py: 100 Patient years
aOR: Adjusted odds ratio
ART: Anti-retroviral therapy
aTP: Active *Treponema pallidum* infection
CI: Confidence interval
CT: Chlamydia trachomatis
df: Degrees of freedom
HIV: Human immunodeficiency virus
IQR: Interquartile range
MSM: Men who have sex with men
VLbLOD: Months viral load below level of detection
NG: Neisseria gonorrhea
OR: Odds ratio
PrEP: Pre-exposure prophylaxis
pTP: Past *Treponema pallidum* infection
SE: Standard error
STI: Sexually transmitted infection

## References

[1] Bremer V, Dudareva-Vizule S, Buder S, An Der Heiden M, Jansen K (2017). Sexuell übertragbare Infektionen in Deutschland. Bundesgesundheitsbl.

[2] Jansen K, Schmidt AJ, Drewes J, Bremer V, Marcus U (2016). Increased incidence of syphilis in men who have sex with men and risk management strategies, Germany, 2015. Eurosurveillance.

[3] Jansen K (2020). Syphilis in Deutschland im Jahr 2019. Neuer Höchststand von Infektionen.

[4] Marcus U, Schmidt AJ, Hamouda O, Marcus U, Schmidt AJ, Hamouda O (2011). HIV serosorting among HIV-positive men who have sex with men is associated with increased self-reported incidence of bacterial sexually transmissible infections. Sex Health.

[5] Jansen K, Steffen G, Potthoff A, Schuppe A-K, Beer D, Jessen H (2020). STI in times of PrEP: high prevalence of chlamydia, gonorrhea, and mycoplasma at different anatomic sites in men who have sex with men in Germany. BMC Infect Dis.

[6] Farfour E, Dimi S, Chassany O, Fouéré S, Valin N, Timsit J (2021). Trends in asymptomatic STI among HIV-positive MSM and lessons for systematic screening. PLoS ONE.

[7] Dudareva-Vizule S, Haar K, Sailer A, Wisplinghoff H, Wisplinghoff F, Marcus U (2014). Prevalence of pharyngeal and rectal *Chlamydia*
*trachomatis* and *Neisseria*
*gonorrhoeae* infections among men who have sex with men in Germany. Sex Transm Infect.

[8] Spinner CD, Boesecke C, Jordan C, Wyen C, Kümmerle T, Knecht G (2018). Prevalence of asymptomatic sexually transmitted infections in HIV-positive men who have sex with men in Germany: results of a multicentre cross-sectional study. Infection.

[9] Nieuwenburg SA, Sprenger RJ, van der Loeff MFS, de Vries HJC (2021). Clinical outcomes of syphilis in HIV-negative and HIV-positive MSM: occurrence of repeat syphilis episodes and non-treponemal serology responses. Sex Transm Infect.

[10] Grewal R, Allen VG, Gardner S, Moravan V, Tan DHS, Raboud J (2017). Serosorting and recreational drug use are risk factors for diagnosis of genital infection with chlamydia and gonorrhoea among HIV-positive men who have sex with men: results from a clinical cohort in Ontario. Canada Sex Transm Infect.

[11] Patel P, Bush T, Mayer K, Milam J, Richardson J, Hammer J (2012). Routine brief risk-reduction counseling with biannual STD testing reduces STD incidence among HIV-infected men who have sex with men in care. Sex Transm Dis.

[12] DAIG, Öag (2018). S2k-Deutsch-Österreichische Leitlinien zur HIV-Präexpositionsprophylaxe.

[13] Sexual transmitted infections 2008 counselling, diagnostic and treatment guideline of the German STI Society. 2022. https://www.awmf.org/leitlinien/detail/ll/059-006.html. Accessed 16 March 2021.

[14] European AIDS Clinical Society. EACS Guidelines Version 10.0. 2022.

[15] Tong CM, Heudebert JP, Tamhane A, Hook E, Van Wagoner N, Dionne-Odom J (2014). 1584 Gonorrhea and Chlamydia testing in routine clinical care of HIV-infected men who have sex with men. Open Forum Infect Dis.

[16] Landovitz RJ, Gildner JL, Leibowitz AA (2018). Sexually transmitted infection testing of HIV-positive medicare and medicaid enrollees falls short of guidelines. Sex Transm Dis.

[17] Berry SA, Ghanem KG, Mathews WC, Korthuis PT, Yehia BR, Agwu AL (2015). Gonorrhea and Chlamydia testing increasing but still lagging in HIV clinics in the United States. J Acquir Immune Defic Syndr.

[18] Burchell AN, Grewal R, Allen VG, Gardner SL, Moravan V, Bayoumi AM (2014). Modest rise in chlamydia and gonorrhoea testing did not increase case detection in a clinical HIV cohort in Ontario, Canada. Sex Transm Infect.

[19] Bernstein KT (2015). Systems approaches to improving rates of extragenital chlamydia and gonorrhea screening among men who have sex with men engaged in human immunodeficiency virus care. Sex Transm Dis.

[20] Barbee LA, Dhanireddy S, Tat SA, Marrazzo JM (2015). Barriers to bacterial STI testing of HIV-infected men who have sex with men engaged in HIV primary care. Sex Transm Dis.

[21] Mark H, Irwin K, Sternberg M, Anderson L, Magid D, Stiffman M (2008). Providers’ perceived barriers to sexually transmitted disease care in 2 large health maintenance organizations. Sex Transm Dis.

[22] Mueller MC, Walentiny C, Seybold U, Nöstlinger C, Platteau T, Borms R (2013). Sexual and reproductive health services for people living with HIV/AIDS in Germany: are we up to the challenge?. Infection.

[23] Taylor MM, Frasure-Williams J, Burnett P, Park IU (2016). Interventions to improve sexually transmitted disease screening in clinic-based settings. Sex Transm Dis.

[24] Botes LP, McAllister J, Ribbons E, Jin F, Hillman RJ, Botes LP (2011). Significant increase in testing rates for sexually transmissible infections following the introduction of an anal cytological screening program, targeting HIV-positive men who have sex with men. Sex Health.

[25] Fuchs W, Kreuter A, Hellmich M, Potthoff A, Swoboda J, Brockmeyer NH (2016). Asymptomatic anal sexually transmitted infections in HIV-positive men attending anal cancer screening. Br J Dermatol.

[26] Statistisches Landesamt Baden-Württemberg. Eckdaten zur Bevölkerung. 2017. https://www.statistik-bw.de/BevoelkGebiet/Bevoelkerung/99025010.tab?R=LA. Accessed 26 Oct 2021.

[27] Khan MA (2012). Blips and its clinical relevance in HIV patients on treatment. Int J Collab Res Internal Med Public Health.

[28] Mueller MC, Usadel S, Kern WV, Zirlik A, Zhou Q (2021). Proportion of patients eligible for statin therapy substantially varies between different cardiovascular disease risk calculators and guidelines used. Int J STD AIDS.

[29] R Core Team. R: a language and environment for statistical computing. 2021.

[30] Keaveney S, Sadlier C, O’Dea S, Delamere S, Bergin C (2014). High prevalence of asymptomatic sexually transmitted infections in HIV-infected men who have sex with men: a stimulus to improve screening. Int J STD AIDS.

[31] Schmidt AJ, Marcus U (2011). Self-reported history of sexually transmissible infections (STIs) and STI-related utilization of the German health care system by men who have sex with men: data from a large convenience sample. BMC Infect Dis.

[32] van der Helm JJ, Hoebe CJPA, van Rooijen MS, Brouwers EEHG, Fennema HSA, Thiesbrummel HFJ (2009). High performance and acceptability of self-collected rectal swabs for diagnosis of *Chlamydia*
*trachomatis* and *Neisseria*
*gonorrhoeae* in men who have sex with men and women. Sex Transm Dis.

[33] Workowski KA, Bachmann LH, Chan PA, Johnston CM, Muzny CA, Park I (2021). Sexually transmitted infections treatment guidelines. MMWR Recomm Rep.

[34] Berry SA, Ghanem KG, Page KR, Gange SJ, Thio CL, Moore RD (2011). Increased gonorrhoea and chlamydia testing did not increase case detection in an HIV clinical cohort 1999–2007. Sex Transm Infect.

[35] Farley TA, Cohen DA, Wu S-Y, Besch CL (2003). The value of screening for sexually transmitted diseases in an HIV clinic. J Acquir Immune Defic Syndr.

[36] Mayer KH, O’Cleirigh C, Skeer M, Covahey C, Leidolf E, Vanderwarker R (2010). Which HIV-infected men who have sex with men in care are engaging in risky sex and acquiring sexually transmitted infections: findings from a Boston community health centre. Sex Transm Infect.

[37] Rosser BRS, Horvath KJ, Hatfield LA, Peterson JL, Jacoby S, Stately A (2008). Predictors of HIV disclosure to secondary partners and sexual risk behavior among a high-risk sample of HIV-positive MSM: results from six epicenters in the US. AIDS Care.

[38] Goethe VE, Angerer H, Dinkel A, Arsov C, Hadaschik B, Imkamp F (2018). Concordance and discordance of sexual identity, sexual experience, and current sexual behavior in 45-year-old men: results from the german male sex-study. Sex Med.

[39] Visser M, Heijne JCM, Hogewoning AA, van Aar F (2017). Frequency and determinants of consistent STI/HIV testing among men who have sex with men testing at STI outpatient clinics in the Netherlands: a longitudinal study. Sex Transm Infect.

