# Supplementary material for: Long-term follow-up after introduction of a systematic sexually transmitted infection screening program for men having sex with men living with HIV in a primary care setting: uptake, STI incidence, and risk factors for infection and reinfection
Source: Infection. 2022 Nov 9;51(4):897–907. doi: 10.1007/s15010-022-01946-0 (PMC10352398; doi:10.1007/s15010-022-01946-0)
Supplement: Supplementary file 1 — Supplementary file1 (DOCX 950 kb) [file 15010_2022_1946_MOESM1_ESM.docx]

# **Supplementary**

Supplementary Figure 1: Heatmap of performed tests by patients and quartal according to result


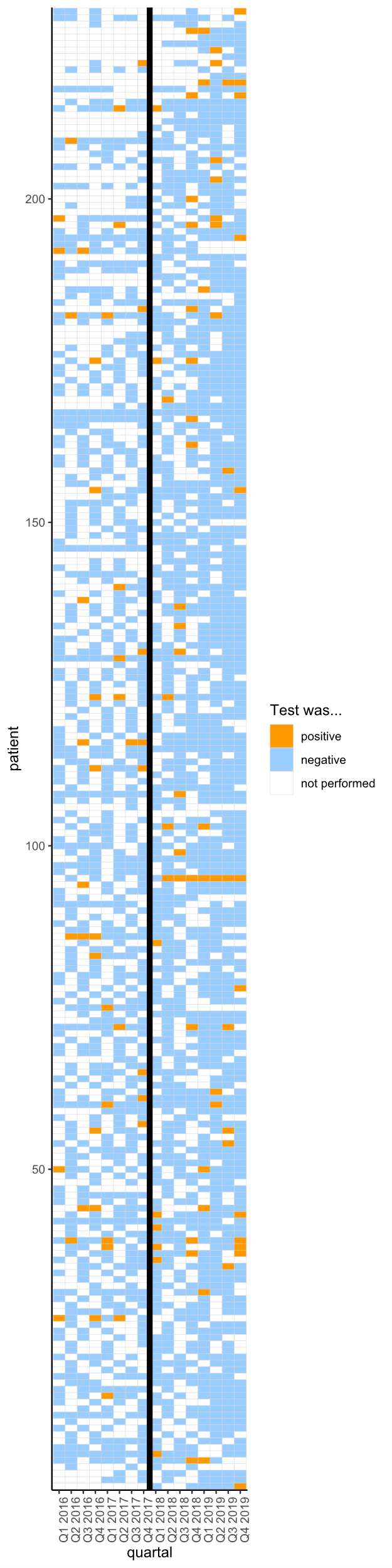


The figure shows the time sequence of performed tests by patient and quartal according to the result for *Chlamydia trachomatis*, *Neisseria gonorrhea* or active *Treponema pallidum* infection (orange = positive; blue = negative; white = not tested). Tiles were colored if patients were tested against at least one of the three pathogens.
